# Pharmacovigilance in perspective: drug withdrawals, data mining and policy implications

**DOI:** 10.12688/f1000research.21402.1

**Published:** 2019-12-16

**Authors:** Muaed Alomar, Subish Palaian, Moawia M. Al-tabakha

**Affiliations:** 1 Clinical Sciences Department, Ajman University, Ajman, Ajman, United Arab Emirates; 2 Pharmaceutical Sciences Department, College of Pharmacy and Health Sciences, Ajman University, Ajman, United Arab Emirates

**Keywords:** Pharmacovigilance, data mining, drug withdrawals, pharmacovigilance policies

## Abstract

Considering that marketed drugs are not free from side effects, many countries have initiated pharmacovigilance programs. These initiatives have provided countries with methods of detection and prevention of adverse drug reactions at an earlier stage, thus preventing harm occurring in the larger population. In this review, examples of drug withdrawals due to effective pharmacovigilance programs have been provided with details. In addition, information concerning data mining in pharmacovigilance, an effective method to assess pharmacoepidemiologic data and detecting signals for rare and uncommon side effects, is also examined, which is a method synchronized with information technology and advanced electronic tools. The importance of policy framework in relation to pharmacovigilance is discussed in detail, and country experiences upon implementation of pharmacovigilance policies is highlighted.

## Introduction

Pharmacovigilance (PV) has been a valuable method in identifying adverse drug reactions (ADRs) and improving the safe use of medicines ^1^. PV has been the backbone for many drug safety interventions, such as drug withdrawals, labelling changes and prescription restrictions ^2– 4^. The advancement and synchronization of information technology had led to valuable contributions in signal detection and data mining processes in PV ^5^. It is important to have policy framing to incorporate PV measures in every country’s drug regulatory mechanisms, so as to implement and sustain drug safety monitoring processes. In this review article, three important aspects of PV are discussed; significant examples of drug withdrawals as an outcome of PV data, data mining and its role in PV, and policy implications related to PV. In addition, PV experiences in selected countries are detailed.

## Examples of drug withdrawals as a result of pharmacovigilance

Many medications have been withdrawn from the market due to their severe, harmful or life-threatening effects. Following marketing approval, once the first ADRs are reported, the reports will be analyzed and the incident will be investigated; and if post marketing surveillance indicates harmful effects for the medication, it will be withdrawn from the market ^6^.

Rofecoxib (Vioxx), manufactured by Merck & Co. in 1999, was indicated as an NSAID in the treatment of “osteoarthritis, rheumatoid arthritis, acute pain and menstrual pain”. In the marketing stages, the company did not mention any cardiovascular disorders. Between 2000 and 2002, reports started to emerge on the hazards of Vioxx, but it took a 3-year clinical trial, “APPROVe” (Adenomatous Polyp Prevention of Vioxx), executed by Merck Frosst Canada, which lead to participants experiencing cardiovascular events such as heart attacks and strokes, for the drug to be withdrawn from the market ^7^. In 2004, during a US Senate hearing concerning rofecoxib issues, Dr. David Graham, who was the associate director in the U.S. Department of Food and Drug Administration (FDA)’s Office of Drug Safety, stated: “The approval of rofecoxib (Vioxx) by the US FDA has led to the single greatest drug safety catastrophe in the history of this country or the history of the world”. It has been estimated that 88,000 to 139,000 Americans have had heart attacks or strokes due to Vioxx. The drug was finally discontinued in 2004 ^8^. 

Lysergic acid diethylamide (LSD), was discovered in 1938 by Dr. Hofmann, who was in Sandoz Laboratories in Switzerland ^9^. After five years, it became evident that the drug was causing hallucinations, euphoria, delusions, depression, as well as suicidal thoughts ^10, 11^. In 1970, LSD was placed in Schedule 1 category of drugs; the most restricted category, following passage of the Controlled Substances Act. This category indicates “a high potential for abuse, no recognized medical use, and no safety when used by a physician” ^9^.

Benfluorex (Mediator) was first manufactured and marketed by Servier in 1976 in France as an add-on therapy for hyperlipidemia and diabetes associated with obesity. In 1998, an official PV investigation was opened regarding the drug in France due to its “potential danger”, and Italian regulators expressed apprehension to the European Medicines Agency (EMA). In 1999, two cardiovascular complications were reported in France. In 2003, Spanish regulators reported a cardiac valvulopathy case to the EMA ^12^. Servier decided to withdraw the drug from Spain and Italy through not renewing their expired license ^13^. In 2009, AFSSAPS (Agence Française de Sécurité Sanitaire des Produits de Santé), the regulatory authority of France ^14^, suspended benfluorex marketing due to “efficacy and safety issues” especially after a case control study that was done by a chest physician, Irene Frachon, in which she discovered that drug-induced valvular heart disease is associated with benfluorex. Servier withdrew the medication worldwide after earning 20 million Euros each year for the previous 15 years ^12^. EMA fully withdrew the drug in 2010 ^13^.

Sibutramine (Meridia, US; Reductil, UK) ^8^, a weight management and weigh loss agent, was approved in Europe in 1999 ^15^, and in other parts of the world. Since 2002, many cardiovascular events were reported, including hypertension, tachycardia, arrhythmia and myocardial infarction (MI). In Sibutramine Cardiovascular Outcomes Trial (SCOUT), results demonstrated that patients with preexisting cardiovascular disease who had taken sibutramine had an increased likelihood of developing MI or stroke ^16^. David Graham - in his testimony before the Senate committee about medications that may harm the patients - included Meridia as one of them ^8^. Sibutramine was withdrawn from the US and European markets in 2010 ^15, 16^.

Pergolide (Permax) was developed by Eli Lilly & Co., and approved in 1988 for the management of Parkinson’s disease symptoms (Reuters, 2007) ^17^. It was then withdrawn from the US market in 2007 due to “increased rates of cardiac valvular dysfunction (cardiac valvulopathy)” ^6^.

Pemoline (Cylert) was approved in Europe in the sixties, and in the US in 1975, for the treatment of attention deficit hyperactivity disorder (ADHD). Between 1975 and 1989, the FDA received reports of “12 cases of jaundice and 6 deaths in youths ascribed to pemoline hepatotoxicity”. Serious hepatotoxicity was unveiled only in 1996. Pemoline hepatotoxicity reporting was not sufficient in the 1980s to stir an action, due to the poor post marketing surveillance and reporting systems ^18^. Following those incidences, the FDA mandated a black box warning to highlight the risk of hepatotoxicity. Afterwards, another case of liver failure was reported, forcing the manufacturer of Cylert (Abbott Laboratories) to cease production in May 2005, and the FDA notified healthcare professionals about the discontinuation of all pemoline products ^19^.

The Contergan scandal took place in early 1960s. Contergan had thalidomide as the active ingredient, a product of a German company “Chemie-Grünenthal”, having sedative effects, apparently non-toxic, with few side effects ^20^. It was used as a sedative, hypnotic and antiemetic for pregnant women ^21^. Mothers who used thalidomide in their early pregnancy had horrific results, as over 10,000 children were born with birth defects such as phocomelia ^22^. It was withdrawn from most markets between 1961 and 1962 ^21^.

Valdecoxib (Bextra) was an NSAID used for arthritis and joint pain ^23^. According to The New England Journal of Medicine, increased cardiovascular events occurred after coronary artery bypass grafting surgery associated with valdecoxib usage ^24^. Cardiovascular complications were reported, as well as “Stevens–Johnson syndrome, erythema multiforme, and toxic epidermal necrolysis”. These reports led to valdecoxib’s withdrawal from the market in 2005 ^23^.

Levamisole (Ergamisol) is an immunomodulatory agent used as an adjunct chemotherapeutic drug. It was manufactured by Janssen Pharmaceutica in 1966. Serious side effects were discovered, including: “neutropenia, agranulocytosis, cutaneous vasculopathy, and leukoencephalopathy” ^25^. Levamisole was withdrawn from the US market in 2000. However, it is currently being used as an antihelminthic in veterinary medicine and can be found in street drugs as a cocaine adulterant ^26^.

Hydromorphone hydrochloride extended release (Palladone) manufactured by Purdue Pharma and launched in the USA in January 2005, was used as a narcotic analgesic ^27^. The FDA withdrew the drug in July of the same year due to dose dumping with alcohol, which leads to accidental overdosing ^27, 28^. Dose dumping is the release of large amount of hydromorphone drug from the extended release form leading to toxicity ^28^.

Cisapride (Propulsid), manufactured by Janssen Pharmaceutica ^29^, was indicated as a prokinetic for severe heartburn associated with gastroesophageal reflux disease. Post marketing studies showed patients experiencing “palpitations, unusual tachyarrhythmia, torsades de pointes, ventricular fibrillation, QT prolongation, and sudden death” ^30^. Cisapride was associated with 341 heart rhythm abnormalities cases and 80 deaths. Most of these cases involved patients taking other medications or have medical conditions that increased cardiac arrhythmia risk ^29^. It was later withdrawn in 2000 ^30^.

Drotrecogin alfa (Xigris) is a recombinant human activated protein C that has anti-thrombotic, profibrinolytic and anti-inflammatory activity ^31^. It was indicated for treatment of severe sepsis. Xigris was marketed by Eli Lilly; the FDA approved it in 2001 ^31, 32^, while the EMA approved it in 2002 ^32^. Following a study (PROWESS-SHOCK) that included 1696 patients and concluded that Drotrecogin alfa did not reduce mortality within 28 days, Eli Lilly voluntarily withdrew Xigris from the market in October 2011, and the FDA and EMA communicated the decision to healthcare professionals ^33, 34^.

Aprotinin (Trasylol), manufactured by Bayer in 1993, was indicated as antifibrinolytic to reduce blood loss during heart surgery ^35^. By the end of 2007, aprotinin was discontinued globally, following Blood Conservation using Antifibrinolytics Trial (BART) findings that suggested an increase in 30-day mortality with aprotinin ^36^. However, in 2012, the EMA recommended that the suspension be lifted ^37^. However, these claims were disputed in another study in 2013, which found that aprotinin may increase the likelihood of mortality in low and intermediate cardiac surgery patients. The study suggested that the decision by the EMA to reinstate the drug for lower risk patients should be debated ^36^.

## Data mining in pharmacovigilance

Data mining is the process of collecting and analyzing data from sources of information that may be raw and complicated (such as data sets or databases) and extracting patterns of links and relationships between these data, to be translated into useful information ^38^. Data mining has been used in many aspects; most importantly it has contributed to drug discoveries, prediction ^39^, diagnosis of diseases (such as diabetes) ^40^ in addition to drug complications and ADRs ^41^. According to Wilson *et al.* ^42^ “Data mining encompasses a number of statistical techniques including cluster analysis, link analysis, deviation detection and disproportionality assessment which can be utilized to determine the presence of and to assess the strength of ADR signals”. Whenever we predict these ADRs, we can reduce the morbidity and mortality rates ^43^.

### Examples of data mining in pharmacovigilance

Many studies have been published using data mining; for instance, in a data mining study to examine the relationship between antipsychotic drugs and myocarditis and cardiomyopathy using Bayesian statistics and found that myocarditis and cardiomyopathy were reported rarely as suspected ADRs, accounting for less than 0.1% (2121) of almost 2.5 million reports ^44^. Furthermore, a study on benzodiazepines using data mining revealed the existence of potential signals for benzodiazepine-associated skin and subcutaneous tissue disorders ^45^.

### Limitations of data mining in pharmacovigilance

Unfortunately, there are factors that affect the prediction of ADRs negatively. For instance, missing data is a major obstacle facing researchers worldwide, particularly with old cases. The issue of missing data is recognized, and several methods have been proposed and studied by researchers to fill the gap, like omitting records with missing information, or computerized modification of the data. However, these methods had their own limitations ^46^. Underreporting of ADRs by healthcare professionals has a negative effect on data mining related to PV, especially for non-serious ADRs. Indeed, when the database is richer with reports and information related to “ordinary” ADRs, the mining process will be optimized; so that non-serious ADRs will act as a “background” against which critical ADRs will be prominent.

Duplication in the reported cases, as well as duplicate information in the databases, are other impactful weakness in the data mining process.

The extracted information through data mining algorithms are not necessarily accurate and precise, so they must be evaluated clinically and dealt with cautiously before any decision is taken ^46^.

### Future of data mining in pharmacovigilance

The use of data mining in the PV of drugs has so far proven effective. Whether examining drugs with non-serious side effects or those that have been withdrawn from the market, data mining has been shown to be a valuable resource in the PV field ^47^ and is currently implemented in the usual procedures of the major regulatory authorities and PV centers ^48^.

With the growing popularity of social media globally, screening social networking sites are probably going to be a standard PV procedure. Therefore, utilization of data mining is likely to expand to mining social data for PV purposes ^49^.

The aims of PV are well-recognized. It has been defined by the World Health Organization (WHO) as “the science and activities relating to the detection, assessment, understanding and prevention of adverse effects or any other drug-related problem”. The main focus of PV is to improve patient safety regarding the rational and safe use of medicine ^50^. It also requires clinical staff to have competence in PV practices ^51^. Furthermore, it is concerned with the early detection of unknown ADRs and identifying risk factors and causes of the ADRs ^49^. 

PV, although a relatively new term that appeared in the seventies of the past century ^52^, is not a new concept. In 1848, a 15-year-old female patient received chloroform as an anesthetic before treatment for an ingrown toenail. The patient developed ventricular fibrillation, which resulted in her death. In 1893, the Lancet published the results of a commission it established in Britain to report ADRs and deaths related to anesthesia. This is considered to be the prototype for an ADR spontaneous reporting system ^53^. 

The most notable event in PV appeared in 1961, when William Mc Bride, an Australian obstetrician, reported that in patients receiving thalidomide to treat morning sickness, up to a 20% surge in the development of fetal malformation was observed ^53^. In the Netherlands, in a study by Lely in 1971, he reported the death of at least 19 people as a result of digitalis intoxication, which was due to an error in the production of the digitalis tablets ^54^. Moreover, in 1974, after four years of marketing practolol in the UK, it was found to cause oculomucocutaneous syndrome and sclerosing peritonitis. Benoxaprofen was withdrawn from the market in 1982, only two years following marketing approval, due to multiple reports of photosensitivity and serious hepatotoxicity. These cases highlighted the importance of recording, reporting and publishing all ADRs. Such reports shed the light on less frequent ADRs and assist healthcare professionals in managing uncommon ADRs, which can be done by doing a quick database search for any similar cases ^55^.

## Policies and procedures for implementing pharmacovigilance

The WHO considers the monitoring of safety and effectiveness of medicines in any country as the responsibility of national governments. The fulfillment of this responsibility can be achieved by establishing national PV centers with well-defined PV systems. The foundation of the launch of PV centers is the establishment of policies regarding the costs, budget and the financial requirements of running the centers ^56^. Financing for a PV center must be secured and given official approval, in order to guarantee the progress of work. The costs incurred depend on the size of population that is serviced by the center and the rate of reports generated. Possible sources of funding may be: insurance companies, academic institutions, and governmental bodies that are interested in the safety of medicinal products ^57^. The location of the center may initially be within a country’s main hospital and, over time, be extended to multiple hospitals all over the country. Each smaller center would then send its reports to the main national center, which would be responsible for gathering the information and communicating and coordinating between the multiple centers. Finally, the main center would conduct and transfer the gathered information to the global PV institutions, such as Uppsala Monitoring Centre (UMC) in Sweden ^57^.

However, this will not be achieved unless the center has a sufficient number of qualified and trained personnel. The WHO determined that the minimum requirement of manpower to work in the PV center is at least one full-time employee ^58^. Staff should give support and be involved in the PV process depending on their assigned tasks and responsibilities. However, these tasks and all organization resources should be structured and arranged to assist in the proper conduct of PV activities. Besides, the presence of qualified leadership is one of the most important factors in order to implement PV techniques and to motivate all staff to achieve the objectives. A good PV system must have excellent data collection methods to gather evidence on the risk/benefit balance and all criteria related to the safety of medicinal products, which will affect decision making. The system must include preparedness plans with appropriate instructions for urgent cases. The center should contain appropriate facilities and equipment which include office space and information technology systems ^59^. Each center should have a database and a standard individual case safety report (ICSR) form and be connected with the national database. The national PV center should recruit an advisory committee, which will help and support the local PV centers in risk assessment and management, and most importantly in crisis ^58^.

As the core of the PV system is to report any ADRs, reports must utilize a specific ADR reporting form and should be unique to each country. The forms should be distributed to all healthcare professionals in all areas, and all healthcare institutions; hospitals, pharmacies, medical centers ^60^. These forms are known as standard ICSR forms ^58^. ICSR is defined in PV as “a notification relating to a patient with an adverse medical event or laboratory test abnormality suspected to be induced by a medicine. It is an essential source of information for the achievement of the main objectives of PV and can involve several ADRs” ^61^. Forms should then be gathered and collected or posted to the center by fax or email to ensure an easy flow of data. The minimum information in these forms should be general information about the patient (such as age, gender, race), a detailed description of the ADR, severity, lab tests (if any), date of appearance of ADR, the suspected medication causing the reported ADR (product information such as brand name, dosage form, the ingredients, and concentration), manufacturer related information, dose of medicine and date of initiation and withdrawal (if applicable), a brief medical history of the patient, the risk factors may be present in the patient like other medical problems or diseases (such as liver or renal problems and allergies), and the name of medical practitioner who detected and observed the ADR ^57^.

For the reporting procedure to be complete, communication of ADR reports to VigiBase, the WHO global database that receives contributions from national PV centers in different countries, is essential for the success of the WHO’s International Drug Monitoring Programme ^56^. The startup of the WHO’s Programme for International Drug Monitoring was in 1968 as a pilot project, with 10 countries already having established national systems for reporting of ADRs. The project then expanded to include more countries all over the world. New member countries developed PV centers to report the ADRs and coordinate with the WHO center in Uppsala, where VigiBase is based. VigiBase contains more than 8 million ADR reports from more than 110 countries ^62^. VigiFlow is an internet-based system that offers free access to all member countries to see all information and reports in VigiBase, and their analysis from all over the world ^63^. In April 2015, the WHO launched VigiAccess, a web application that allows anyone to access information. This is a significant step, which encourages reporting ADRs ^64^.

PV programs and drug regulatory authorities must be linked together so that the regulatory authority is continuously updated about any emerging safety issue, and at the same time regulatory authorities should know the critical need for the PV concept, leading to a focus not only on the approval of new medicines, but on their safety as well ^56^. 

In order to assure reliable PV program, it is crucial to periodically train staff on gathering and analyzing information related to ADRs, risk management, signal detection, data mining and possible actions in cases of a serious or fatal ADRs ^60^. Today’s quick-paced advancing world implies that PV personnel may require training on new skills, technologies and concepts, like artificial intelligence and machine-learning.

Moreover, training should be directed not only for the staff with specific PV tasks but also those with activities that have impact on the PV system, including clinical trials, and regulatory affairs ^59^. *As* the training and education of healthcare professionals in the services of PV increases, patient safety will improve, quality of ADR reports will be enhanced, and the development of policies to prevent them will occur.

A non-randomized study was conducted in a hospital in Brazil in 2012 on a multidisciplinary group of healthcare providers. Educational intervention involved different methods such as: lectures on PV, a practical class about reporting ADRs, and distribution of written materials about PV to healthcare providers. In order to assess the level of information about PV among the participants, a questionnaire was given before and after the intervention. The results showed that the educational intervention was successful in the understanding of PV concepts, and improving skills to effectively complete reports related to ADRs. Directly after the educational intervention, the number of reports increased; however, four months after the educational intervention the number decreased, which implies that the education has to be continuous or periodic to keep the motivation among healthcare practitioners toward reporting ADRs ^65^.

## Implementation of pharmacovigilance regulations: country experiences

### United States of America

After the most famous disaster of thalidomide in 1960's, the US FDA revised their regulations through imposing stricter rules on medicine approvals and establishing a spontaneous reporting PV system to report the ADR incidence in the healthcare sector ^53, 66, 67^.

A meta-analysis of prospective studies was performed from four electronic databases over a period of 32 years, from 1966 to 1996, including 39 studies in US hospitals in order to evaluate the occurrence of serious drug reactions. From these studies, it was found that a large number of hospital patients died from fatal ADRs, which were estimated to be the fourth to sixth leading cause of death in 1994, representing an important clinical issue ^66^.

The FDA regulates PV with the help of the Center for Drug Evaluation and Research (CDER). This center evaluates new drugs before they can be marketed and maintains a rigorous post marketing safety surveillance program, monitoring the use of marketed drugs for unexpected health risks ^68^.

According to the American Society of Health-System Pharmacists (ASHP), ADR reports would vary due to the different size and type of the healthcare centers, patient mix, definition of ADR, and the medications used. The foremost obligation of reporting them is on pharmacists, physicians, nurses and even patients, and should include full information about the incident including the patient's name, patient's history etc. All ADR reports are analyzed and evaluated by a medical committee and in the case of serious ADR reports, they are reported to the FDA or drug's manufacturer or both ^69^.

Most serious ADR reporting, whether voluntary or mandatory, is done through the MEDWatch program, belonging to the FDA. This program was introduced in 1993 ^70^. There are three forms that have been developed by the FDA for the reporting of ADRs: Form FDA 3500, for voluntary reporting by healthcare professionals; Form FDA 3500B, for voluntary reporting by patients and consumers; and Form FDA 3500A, for mandatory reporting ^71^. The regulation and evaluation of ADRs may lead to regulatory action by the FDA, including labeling changes, risk management action plan (educating about the new safety information, and controlling distribution of the drug), removing the drug from the market, or conducting further studies ^72^.

A survey was conducted for a period of four months between May and August 2014 in three U.S. states (New Jersey, New York and Washington) in different health sectors, including pharmacists, nurses and physicians, to evaluate the ADR reporting process, and to explore gaps and issues in the reporting process. Results of the survey showed that the reporting process of ADRs was mainly to FDA MedWatch, internal reporting, and to the drug manufacturer. During the survey, factors for not reporting ADRs were: gaps in technology, gaps in education, and gaps in the overall process. Recommendations for improving the ADR reporting system include: improving integration between electronic systems, increasing awareness by training and educating patients and healthcare providers of the ADR reporting process, and simplifying and initiating a standard ADR-reporting process ^73^.

### United Kingdom

The UK established an ADR monitoring system through spontaneous reporting, post marketing surveillance, and interrogating large databases. Spontaneous reporting of any ADR is carried out through the Yellow Card Scheme. Yellow Card reports are sent by healthcare professionals and patients by mail, telephone or through the internet to the Medicines and Healthcare product Regulatory Agency (MHRA). All reports are gathered and reviewed to detect any safety issue ^74^.

Newly marketed medicines are marked with an inverted black triangle. The triangle indicates that all doctors have to report all ADRs that are detected by patients after using the new medicine, through the Prescription-Event Monitoring technique. All reports are then submitted to the Committee on Safety of Medicines (CSM) ^75^.

### France

The French Agency for the Safety of Health Products, ANSM (Agence nationale de sécurité du médicament etdes produits de santé), is the authority responsible for PV activities implementation and coordination in France ^76^.

ANSM succeeded AFSSAPS (Agence Française de Sécurité Sanitaire des Produits de Santé - French Agency for the Sanitary Safety of Health Products) as the PV regulation authority in 2011. AFSSAPS was heavily criticized for failing to withdraw Mediator (benfluorex) for a very long time, despite numerous reports of severe ADRs, and the fact that it was discontinued in several other countries. Therefore, the French government decided to establish ANSM in order to replace AFSSAPS ^14^.

ANSM coordinates the national PV system, which is integrated into the European PV system. The PV system in France is based upon a network of 31 Regional PV Centers (Les centres régionaux de pharmacovigilance [CRPV]), responsible for monitoring, evaluating and preventing ADRs and risks, and promoting the optimum medicine usage. The CRPVs collect and transfer ADR reports to ANSM, and support healthcare professionals with information about PV. The Technical Pharmacovigilance Committee (Comité tech-nique de pharmacovigilance [CTPV]) comprises of all CRPV directors and ANSM management. CTPV is responsible for recommending “follow-up, analysis and prevention of risks”.

Healthcare professionals may report ADRs to CRPV, or to the pharmaceutical company. Both CRPV and the pharmaceutical company should forward the report to ANSM. Patients now can report ADRs as well, through an online report form ^77^.

### United Arab Emirates

Due to the expansion of the expatriate population of the UAE, the healthcare system is attempting to meet mounting healthcare needs ^78^. The main organizations responsible for the regulation of healthcare in the UAE are the Ministry of Health and Prevention (MOHAP), Department of Health – Abu Dhabi (DOH), and Dubai Health Authority (DHA).

A number of policies and legislations have been drawn up regarding accessibility, availability, affordability, quality, and pricing of medicines; nevertheless, proper application remains a concern ^79^. The UAE. initiated its PV program in 2008 ^79^. It officially joined the WHO International Drug Monitoring Programme in collaboration with the Uppsala Monitoring Centre in 2013 ^80^. 

Studies have been carried out to determine the knowledge, attitude, and practice (KAP) of ADR reporting among healthcare professionals in the UAE to identify their current strategies and pinpoint steps to reduce underreporting. One study found that there was poor KAP among healthcare providers in the UAE. The results showed that 81%, 83%, and 83.3% of doctors, community pharmacists, and hospital pharmacists, respectively, were unaware that the UAE had an ADR reporting center, and 56%, 60%, and 72% did not know the correct procedure for reporting ADRs. As such, it was noted that only 19%, 14%, and 12.1% of doctors, community pharmacists, and hospital pharmacists reported ADRs ^81^. Further studies have shown that 72% of pharmacists and 86.7% of physicians were unaware of the ADR reporting system in UAE ^82^.

## Conclusions

In the past, PV has contributed to identifying the safety of medications at an earlier stage and thus preventing harmful effects of medicines affecting much larger populations. Several drugs have been banned based on the safety findings obtained during PV programs. Data mining is a powerful method of early detection of ADR signals and can provide valuable contribution to PV if properly integrated with modern information technology tools. Several countries have implemented policies governing PV that offered them substantial benefits in terms of ensuring patient safety. 

## Data availability

No data is associated with this article.
