# Supplementary material for: Pharmacovigilance in perspective: drug withdrawals, data mining and policy implications
Source: F1000Res. 2019 Dec 16;8:2109. [Version 1] doi: 10.12688/f1000research.21402.1 (PMC7050268; doi:10.12688/f1000research.21402.1)
Supplement: Peer review history

# Peer review: Reviewer response for version 1

**DOI:** 10.5256/f1000research.23572.r60295

**Published:** 2020-02-28

**Authors:** Mohammed Alshakka

**Affiliations:** 1 Department of Pharmaceutics, Section of Clinical Pharmacy, Faculty of Pharmacy, Aden University, Aden, Yemen

An excellent article in which the authors exerted a great and wonderful effort and discussed an extremely important topic for HCP, patients and health in general:

 I have only two comments which will strengthen the article:

- Please add to your text example for low income countries and add several challenges that face running strong pharmacovigilance activities in developing countries.
- What are the strategies to uplift the current PV program in UAE and developing countries? Strategies such as teaching PV and enhancing patients and consumer reporting etc...

I confirm that I have read this submission and believe that I have an appropriate level of expertise to confirm that it is of an acceptable scientific standard.

# Peer review: Reviewer response for version 1

**DOI:** 10.5256/f1000research.23572.r57875

**Published:** 2019-12-23

**Authors:** Nisha Jha

**Affiliations:** 1 Department of Clinical Pharmacology, KIST Medical College, Lalitpur, Nepal

The article has been written in a very appropriate way. This review article has also used relevant literature and the information given is very appropriate in the field of pharmacovigilance. The references are used accurately. Data miming information about the pharmacovigilance has been considered as an important area of information and this article will help young researchers to work more in this area. Overall, the article has been very well written.

I confirm that I have read this submission and believe that I have an appropriate level of expertise to confirm that it is of an acceptable scientific standard.
